# A meta-analysis on the role of pleiotrophin (PTN) as a prognostic factor in cancer

**DOI:** 10.1371/journal.pone.0207473

**Published:** 2018-11-14

**Authors:** Jiupeng Zhou, Yuanli Yang, Yongfeng Zhang, Heng Liu, Quanli Dou

**Affiliations:** Xi’an Chest Hospital, Xi’an, Shaanxi Province, China; Duke Cancer Institute, UNITED STATES

## Abstract

**Background:**

Some researchers reported that pleiotrophin (PTN) is associated with the development and metastasis of various tumors and it is a poor prognostic factor for the tumor patients. However, the results of other researches are inconsistent with them. It is obliged to do a meta-analysis to reach a definite conclusion.

**Methods:**

The published studies relevant to PTN were searched in the databases including PubMed, Embase and Web of Science until March 20, 2018. A meta-analysis was conducted to evaluate the role of PTN in clinicopathological characteristics and overall survival (OS) of cancer patients.

**Results:**

Our meta-analysis indicated that the high expression of PTN was remarkably associated with advanced TNM stage (OR = 2.79, 95%CI: 1.92–4.06, P<0.00001) and poor OS (HR = 1.77, 95%CI: 1.41–2.22, P<0.00001) in tumor patients. The expression of PTN was not associated with tumor size (OR = 1.12, 95% CI: 0.55–2.26, P = 0.76), lymph node metastasis (LNM) (OR = 1.95, 95%CI: 0.62–6.12, P = 0.25), distant metastasis (DM) (OR = 2.78, 95%CI: 0.72–10.74, P = 0.14) and histological grade (OR = 1.95, 95%CI: 0.98–3.87, P = 0.06).

**Conclusion:**

The high expression of PTN is significantly relevant to the advanced TNM stage and poor OS in tumor patients. PTN can serve as a promising biomarker to predict unfavorable survival outcomes, and it may be a potential target for tumor treatment.

## Introduction

Tumour has become a leading cause of death in recent decades, and about 8.2 million people died from cancers and 14.1 million cancer cases were latest diagnosed all over the world in 2012[1]. Different cancers have different mechanisms of tumor formation, and the precise pathogenesis of cancers is still undefined. However, the latest studies have highlighted that PTN is up-regulated in numerous human malignancies and associated with the occurrence of many human malignant tumors[2–8], including colorectal cancer, glioblastoma, melanoma, pancreatic cancer, breast cancer and lung cancer. For instance, PTN promotes the expression of vascular endothelial growth factor (VEGF) and the angiogenesis of colorectal cancer[2]. PTN also boosts the occurrence of glioma induced by platelet-derived-growth-factor-B via promoting the proliferation of neural precursor cells[4]. Moreover, some studies have demonstrated that the PTN expression is associated with certain clinical characteristics, such as lymph node metastasis (LNM), distant metastasis (DM), clinical stages and overall survival (OS)[4,6,9], while the results of some other researches are conflicting with them[5,7,10]. The aim of this meta-analysis is to explore the prognosis and clinicopathological factors of pleiotrophin (PTN) in tumor patients considering the possible deviations of individual studies.

## Materials and methods

### Literature search strategy

In order to obtain the potential qualified reportes, a systematic network reference search was conducted focusing on multiple website databases, including Embase, PubMed, and Web of Science until March 20, 2018 and the search keywords were as follows: “pleiotrophin”, “PTN”, “cancer”, “tumor”, “clinicopathology”, “prognosis” and “survival”. The relevant reviews and references cited in the searched articles were also filted to avoid leaving out any potentially usable references. Besides, other related articles were also available by examining the reference lists by hand.

### Selected and removed criteria

The selected criteria were as follows: 1) the expression level of PTN in primary cancerous tissues was measured; 2) dichotomous model was appraised by immunohistochemistry (IHC); 3) related clinicopathologic parameters were reported; 4) hazard ratio (HR) and 95% confidence interval (CI) between PTN expression and overall survival could be picked up directly or calculated indirectly in the study. The removed criteria were as follows: 1) the letters, experiments, or articles that researched on animal models; 2) repeated research publications; 3) the expression of PTN was detected in serum or at PTN mRNA level.

### Date extraction and quality evaluation

Two investigators (Zhou J and Yang Y) independently extracted the information and data from all eligible studies through cross-check. The data and information were got together from every study using a purpose-designed form: the author, the year of publication, the country, the type of cancer, the overall number of patients, number of patients in the high PTN expression group and the low PTN expression one, number of patients with big tumor size, TNM (Ⅲ/Ⅳ), LNM, DM and survival data in each group, and the standard for high PTN expression. The survival results of both original and adjusted data were overall survival. The disagreements between the two investigators were settled by discussion until a agreement was reached with the third investigator (Zhang Y). Quality evaluation was based on the Newcastle-Ottawa quality assessment scale (NOS). The NOS scores varied from 0 to 9. Six points or more were deemed as high quality.

### Statistical analysis

The current meta-analysis was carried out using the RevMan5.3 software and Stata SE13.0 software. The prognostic effect of high PTN expression was assessed by both adjusted and unadjusted hazard ratios (HRs) and their 95% confidence intervals of overall survival from the primary studies. According to the standard of each study, the expression of PTN was divided into high and low level groups. Reported HR and 95% CI were directly collected from the researches. If HR and 95% CI were not shown in these studies, the means of Tierney et al.[11] and Parmar et al.[12] were recommended. The heterogeneity among the enrolled studies was performed by I^2^ metric and Q statistic. The P value<0.05 for the Q-test and the I^2^-value>50% were took into account to be indexes of serious heterogeneity. The random-effects model was selected for the researches with a remarkable heterogeneity (P≤0.05, I^2^≥50%). Or else, the fixed-effects model was applied (P>0.05, I^2^<50%). Sensitivity analysis was performed to ensure the steadiness of the combined results. Publication bias was appraised by Egger’s test and a funnel plot, and P<0.05 demonstrated remarkable bias. The P value <0.05 was deemed statistically significant.

## Results

### Studies identification and characteristics of eligible studies

**Fig 1:**
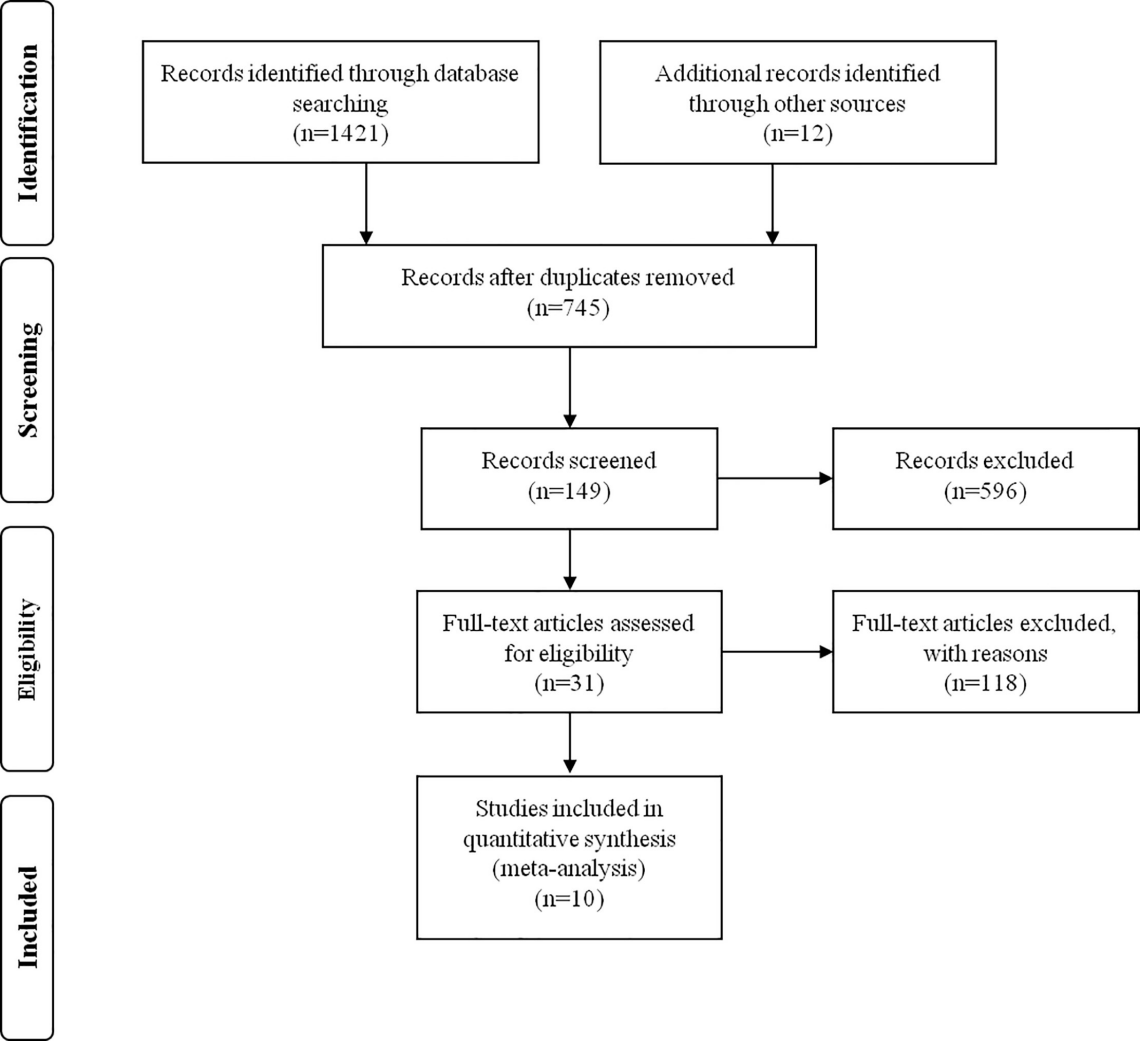
A flowchart presenting the steps of literature retrieval and selection.

After the initial search algorithm, a total of 745 articles were retrieved. Unrelated articles were excluded from headlines and abstracts, and 149 articles were further evaluated in the full text. Those articles, which were Review or Case and did not provided survival data to extract, dichotomous variables and valuable data, were excluded. Finally, this meta-analysis contained 10 articles including a total number of 851 patients (Fig 1). The average sample number of patients every study was 85.1 (range: 34–168). In the 10 studies, seven of them came from the People’s Republic of China, one from Korea, one from U.S.A and one from Sweden. In this meta-analysis, nine different types of cancers were contained, which respectively were one breast cancer, one small cell lung cancer, one cervical cancer, one osteosarcoma, one melanocytic tumor, two glioma, one pancreatic cancer, one colorectal cancer and one hepatocellular cancer (Table 1).

**Table 1:** The basic information and data of all included studies in the meta-analysis.

Author(year)	Country	Cancer type	Total number	PTN expression		Detection method	Criterion of high expression	Quality stars (NOS)	
				High	Low				
**Hye -Sung 2002**	Korea	CC	42	24	18	IHC	The cells stained >25%		7
**H.Wu2005**	USA	MCT	34	20	14	IHC	IRS≥7		7
**Jun Yao2009**	China	PC	38	24	14	IHC	The cells stained >25%		8
**YingKong2012**	China	CRC	83	50	33	IHC	The cells stained >25%		8
**JinyangMaa2014**	China	GM	168	126	42	IHC	IRS≥4		9
**Lei Zhang2015**	Sweden	GM	31	9	22	IHC	The cells stained >0		8
**Lei Zhang2015**		GM	79	36	43	IHC	The cells stained >25%		8
**HQ.Wang2015**	China	LC	83	41	42	IHC	average absorbance value >0.2134		9
**PeisongBai2017**	China	HCC	80	47	33	IHC	The cells stained >25%		8
**DapengWu2017**	China	OC	133	59	74	IHC	IRS≥6		9
**Jiequn Ma2017**	China	BC	80	47	33	IHC	IRS≥6		9

CC, cervical cancer; MCT, melanocytic tumor; pc, pancreatic cancer; CRC, colorectal cancer; GM, glioma; LC, lung cancer; HCC, hepatocellular carcinoma; OC, osteosarcoma; BC, breast cancer.

The association between PTN expression and OS was reported in seven studies[3–7,9,13]. Six articles reported the link of PTN expression and tumor size[2,3,6–9]. Three articles discussed on the relation of PTN expression and lymph node metastasis[6–8]. Four articles discussed on the relation of PTN expression and distant metastasis[5,6,8,13]. Five articles reported the relation of PTN expression and histological grade[2,3,6–8], and seven articles reported the association of PTN expression and TNM stage[2,6–9,13,14] (Table 2). The percentage of dyed cells or existence of nuclear immunoreactivity were identified as the criteria of high/low PTN expression in over half of the studies, while the integrated scoring system (the intensity and percentage of dyed cells) was identified in other studies. If there were both unadjusted and adjusted HRs in the study, the adjusted HRs were adopted in the meta-analysis, if not, the unadjusted HRs contained. The tumor patients in all of the ten researches were separated into two groups (PTN high expression group and PTN low expression group).

**Table 2:** The research results of all included studies in the meta-analysis.

Author(year)	PTN expression	Tumor size		LNM		DM		HG		TNM stage		OS			
		big	small	yes	no	yes	no	H/M	L	Ⅰ/Ⅱ	Ⅲ/Ⅳ	HR	95%CI	In(HR)	Se((InHR))
**Hye -Sung 2002**	High	-	-	-	-	-	-	-	-	17	7	-	-	-	-
	Low	-	-	-	-	-	-	-	-	18	0	-	-	-	-
**H.Wu2005**	High	-	-	-	-	14	6	-	-	-	-	0.698	0.02–20.04	-0.36	1.72
	Low	-	-	-	-	1	13	-	-	-	-	-	-	-	-
**Jun Yao2009**	High	7	17	18	6	4	20	18	6	8	16	1.323	0.34–5.1	0.28	0.69
	Low	0	14	5	9	0	14	6	8	12	2	-	-	-	-
**YingKong2012**	High	29	13	-	-	-	-	23	27	13	37	-	-	-	-
	Low	16	8	-	-	-	-	24	9	16	17	-	-	-	-
**Jiequn Ma2014**	High	99	27	-	-	-	-	34	92	-	-	2.024	1.107–3.695	0.705	0.307
	Low	31	11	-	-	-	-	21	21	-	-	-	-	-	-
**Lei Zhang2015**	High	-	-	-	-	-	-	-	-	-	-	1.878	0.8–4.41	0.63	0.44
	Low	-	-	-	-	-	-	-	-	-	-	-	-	-	-
**Lei Zhang2015**	High	-	-	-	-	-	-	-	-	-	-	1.768	1.2–2.6	0.57	0.2
	Low	-	-	-	-	-	-	-	-	-	-	-	-	-	-
**HQ.Wang2015**	High	27	14	26	15	5	36	24	17	21	20	-	-	-	-
	Low	39	3	30	12	7	35	32	10	30	12	-	-	-	-
**PeisongBai2017**	High	31	16	-	-	-	-	-	-	25	22	1.682	0.96–2.97	0.52	0.29
	Low	21	12	-	-	-	-	-	-	30	3	-	-	-	-
**DapengWu2017**	High	-	-	-	-	21	38	-	-	25	34	1.584	0.982–2.553	0.46	0.244
	Low	-	-	-	-	17	57	-	-	41	33	-	-	-	-
**Jiequn Ma2017**	High	33	14	32	15	-	-	14	33	27	20	2.901	1–8.1	1.065	0.534
	Low	19	14	15	18	-	-	18	15	23	10	-	-	-	-

DM, distant metastases; LNM, lymph node metastases; HG, histological grade; OS, Overall survival.

### Meta-analysis

#### Association between PTN expression and tumor size

**Fig 2:**
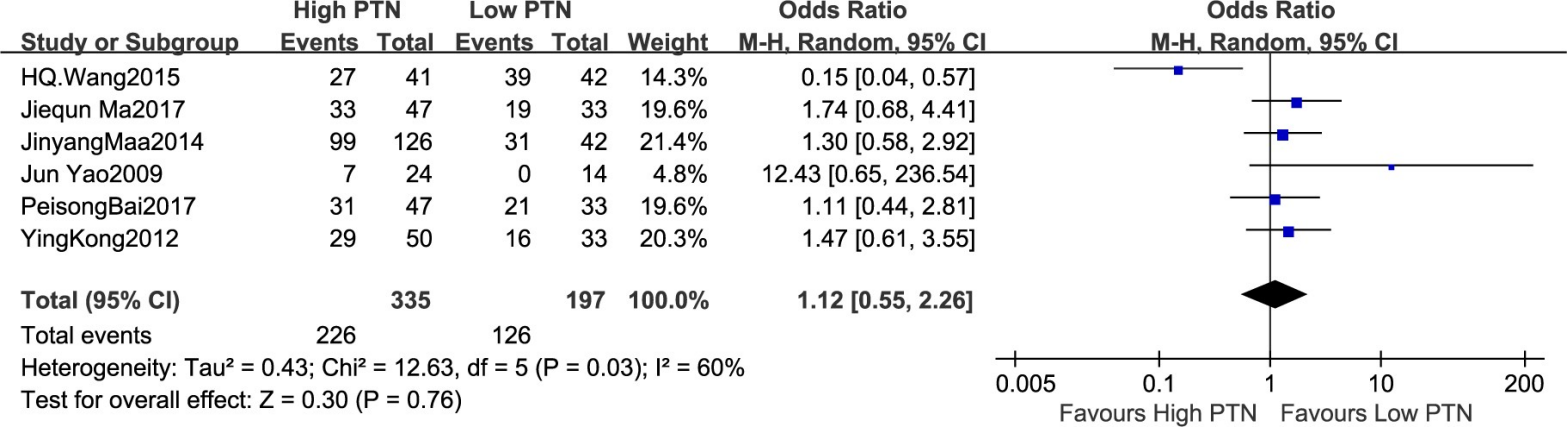
A forest plot for the association between the PTN expression levels with tumour size. The tumor size was not markedly increased in the high PTN expression group compared with the low PTN expression group.

The number of patients with tumor size was reported in six studies in view of different PTN expression levels, totally including 532 patients. There existed notable heterogeneity in the studies (I^2^ = 60%, P = 0.03), thus the random-effects model was employed. The analysis displayed a pooled OR = 1.12 (95% CI: 0.55–2.26, P = 0.76), as shown in Fig 2. The tumor size was not markedly increased in the high PTN expression group compared with the low PTN expression group. Owing to the relatively severe heterogeneity among the studies on tumor size, the sensitivity analysis was carried out. We investigated the impact of individual study on the overall risk assessment by excluding a single study at one time. The pooled OR estimates were consistent without distinct fluctuation, with a scope from 1.0 (95% CI: 0.50–1.98, P = 1.00) to 1.45 (95% CI: 0.94–2.24, P = 0.10). This analysis approved the stabilization of our results.

#### Association between PTN expression and LNM

**Fig 3:**
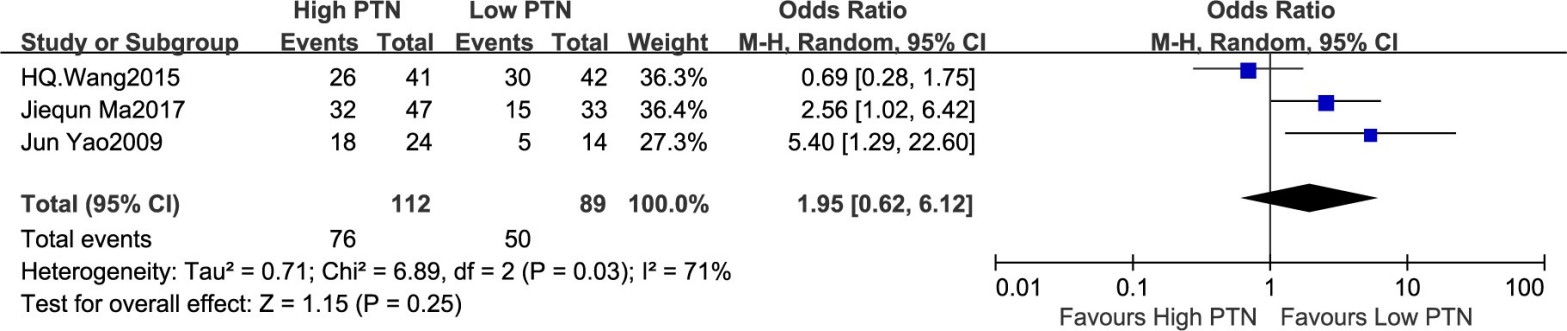
A forest plot for the association between the PTN expression levels with LNM. The LNM was not remarkably increased in the high PTN expression group compared with the low PTN expression group.

Three studies discussed the number of patients with LNM in according to different PTN expression levels, which included 201 patients. The random-effects model was applied because of significant heterogeneity among the studies (I^2^ = 71%, P = 0.03). The meta-analysis indicated a pooled OR = 1.95 (95% CI: 0.62–6.12, P = 0.25), as shown in Fig 3. The LNM was not remarkably increased in the high PTN expression group compared with the low PTN expression group. On account of the relatively big heterogeneity among the studies on LNM, the sensitivity analysis was carried out, in which one study at a time was deleted and the others were analysed to estimate whether the results could have been impacted markedly by one study. Sensitivity analysis showed that the Wang 2015 study was the origin of statistical heterogeneity in the meta-analysis. There existed no evidence of heterogeneity in the left studies (P = 0.39, I^2^ = 0%) when the study from Wang 2015 was removed. The pooled OR estimates were significantly changed to 3.18 (95% CI: 1.47–6.90, P = 0.003). Thus, the LNM was significantly related to the high PTN expression. This analysis confirmed the instability of our results which needed to be treated with caution.

#### Association between PTN expression and DM

**Fig 4:**
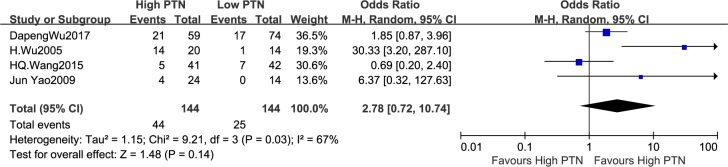
A forest plot for the association between the PTN expression levels with DM. There was not notable difference in the DM incidence between the high PTN and low PTN expression group.

It was reported that the number of patients with DM on account of different PTN expression levels in four articls, which consisted of 288 patients. In virtue of remarkable heterogeneity in the studies,the random-effects model was put to use (I^2^ = 67%, P = 0.03). The pooled odds ratio (OR) of the high PTN expression group compared with low PTN expression group was 2.78 (95%CI: 0.72–10.74, P = 0.14), as shown in Fig 4. The results displayed that there was not notable difference in the DM incidence between the high PTN and low PTN expression group. Owing to the heterogeneity across studies on DM, the sensitivity analysis was carried out,in which one study at a time was deleted and the others were analysed to estimate whether the results could have been affected remarkably by one study. The pooled OR estimates were consistent without obvious fluctuation,with a range from 1.47 (95% CI: 0.63–3.41, P = 0.37) to 5.66 (95% CI: 0.84–38.10, P = 0.07). This analysis confirmed the stability of our results.

#### Association between PTN expression and histological grade

**Fig 5:**
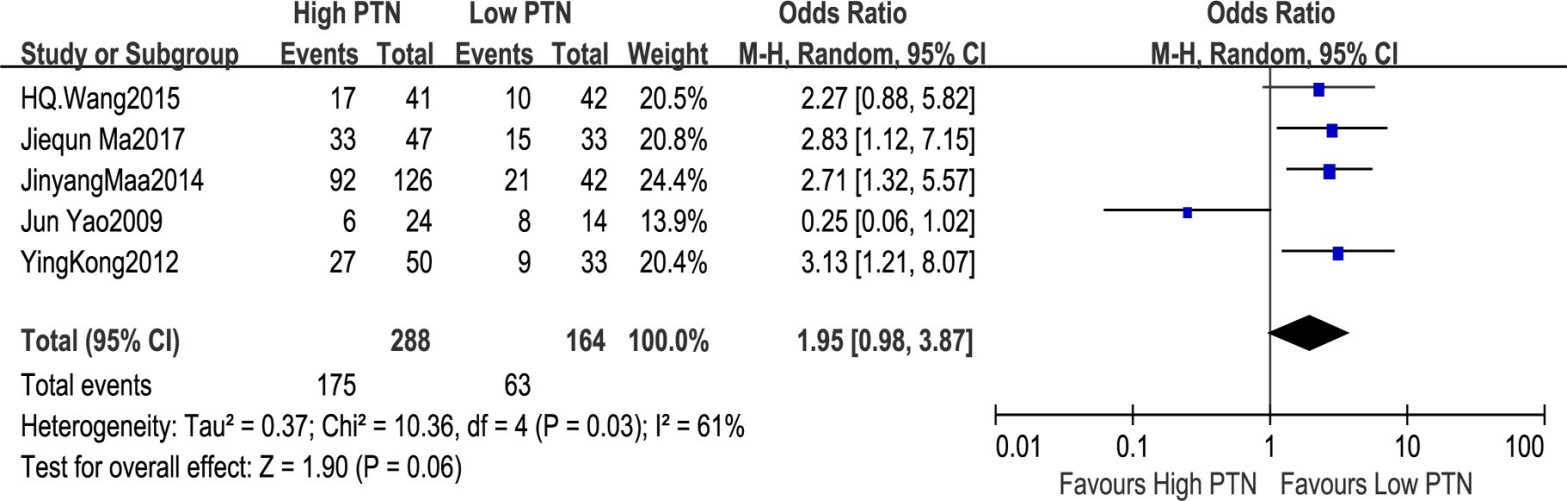
A forest plot for the association between the PTN expression levels with histological grade. The expression of PTN was independent of histology grade.

On the basis of different levels of PTN expression, five studies covered the number of patients with histological grade, including 452 patients. The random-effects model was applied because of significant heterogeneity in the studies (I^2^ = 61%, P = 0.03). The analysis showed a pooled OR = 1.95 (95%CI: 0.98–3.87, P = 0.06), as shown in Fig 5. The results demonstrated that the expression of PTN was independent of histology grade. The sensitivity analysis was carried out owing to the relatively big heterogeneity among the studies on histological grade.Sensitivity analysis revealed that the Yao et al’s study in 2009 was the cause of statistical heterogeneity. When the study from Yao et al’s study in 2009 was removed, the heterogeneity disappeared in the remaining studies (P = 0.97, I^2^ = 0%). The analysis of these left studies indicated a statistically obvious association between the high histological grade and the high PTN expression. The combined OR estimates were 2.71 (95% CI: 1.76–4.19, P<0.00001). This analysis confirmed the instability of our results which needed to be treated with caution.

#### Association between PTN expression and TNM stage

**Fig 6:**
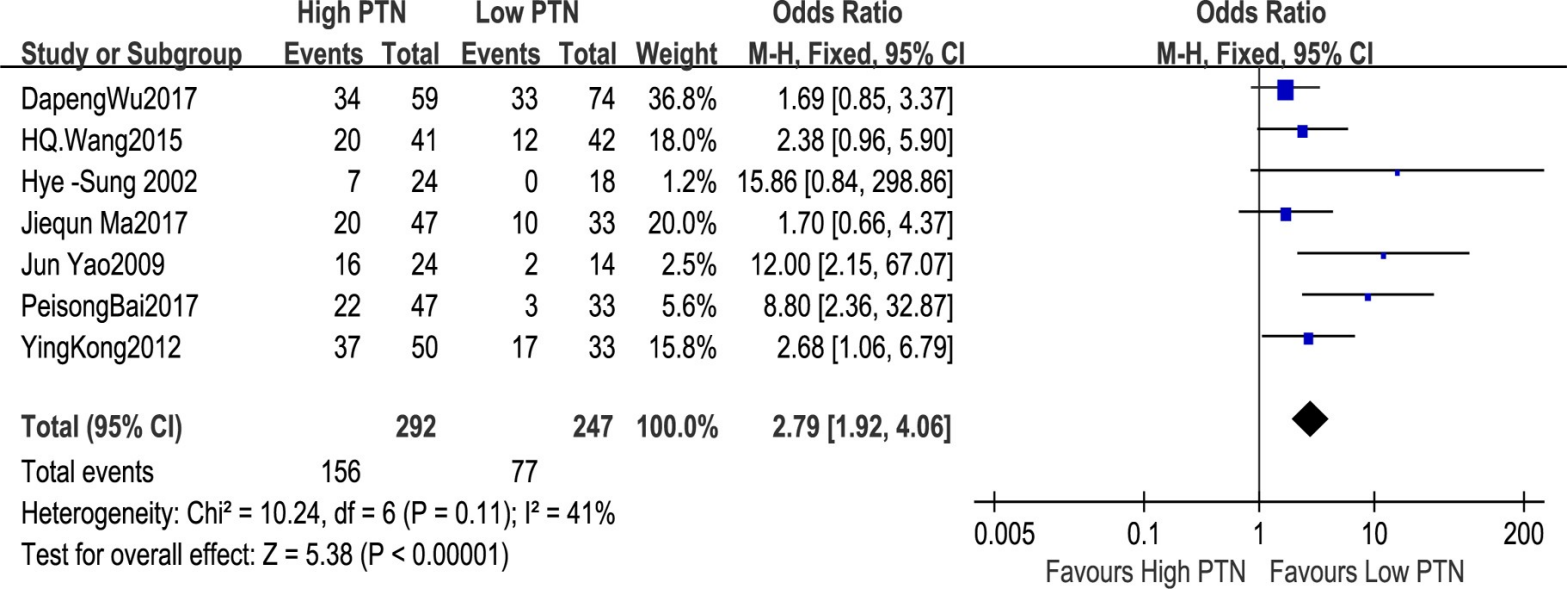
A forest plot for the association between the PTN expression levels with TNM stage. The advanced TNM stage was distinctly related to the high PTN expression.

The number of patients with TNM stage on the basis of different levels of PTN expression was reported in seven studies, including 539 patients. The fixed-effects model was employed because of restricted heterogeneity among the studies (I^2^ = 41%, P = 0.11). The meta-analysis demonstrated a combined OR = 2.79 (95%CI: 1.92–4.06, P<0.00001), as shown in Fig 6. The results displayed that the advanced TNM stage was distinctly related to the high PTN expression. Owing to the relatively limited heterogeneity among studies on TNM stage, the sensitivity analysis was not performed.

#### Association between PTN expression and OS

**Fig 7:**
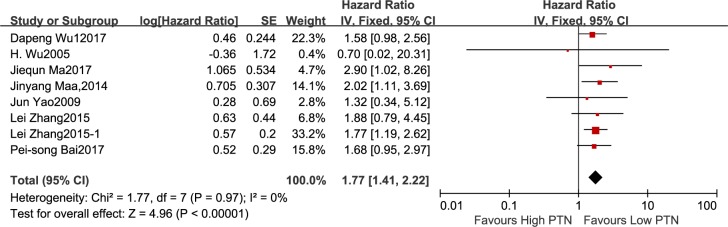
A forest plot for the association between the PTN expression levels with OS. The analysis indicated a poor prognosis in the high PTN expression group.

Seven studies reported 643 patients with OS in the light of different levels of PTN expression. The fixed-effects model was carried out for small heterogeneity in the studies (I^2^ = 0%, P = 0.97). The analysis indicated a pooled HR = 1.77(95%CI: 1.41–2.22, P<0.00001), as shown in Fig 7, demonstrating a poor prognosis in the high PTN expression group. The sensitivity analysis was not employed because of the relatively restricted heterogeneity among studies on OS.

#### Publication bias

**Fig 8:**
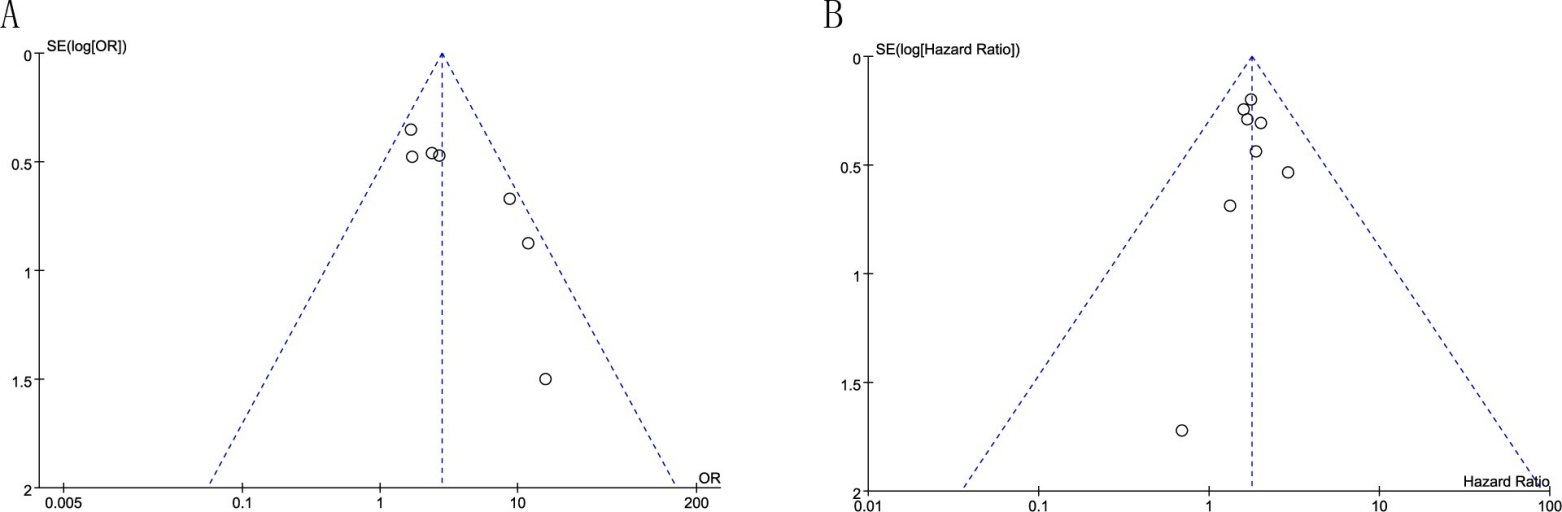
A funnel plot analysis of potential publication bias. **(**A) Egger’s test demonstrated significant publication bias for TNM stage **(**B) There was not publication bias for OS.

Egger’s test was used to evaluate the publication bias. There was not publication bias for tumor size (P = 0.912), LNM (P = 0.544), DM (P = 0.385), HG (P = 0.084) and OS (P = 0.873) (Table 3, Fig 8B) from the studies. Egger’s test demonstrated significant publication bias for TNM stage (P = 0.012) (Table 3, Fig 8A). The bias indicated the presence of a language bias, a potential publication bias, an exaggerated estimates by a flawed methodologic design in smaller sample studies, and a deficiency of publication of small samples with contrary results.

**Table 3:** The publication bias test including literatures.

	Coef	95%CI	P
**Tumor size**	0.273	-6.149–6.694	0.912
**LNM**	5.319	-72.215–82.853	0.544
**DM**	3.984	-31.034–39.001	0.385
**HG**	6.012	-1.507–13.531	0.084
**TNM stage**	-2.933	-4.881- -0.985	0.012
**OS**	-0.072	-1.123–0.980	0.873

## Discussion

PTN, previously designated heparin binding growth-associated molecule, is a multifunctional growth factor that regulates various cellular functions, including cell proliferation, migration, and angiogenesis in endothelial cells, and is a highly conserved member of the human gene families[15]. Human PTN gene is located on chromosome 7, at 7q33, containing nine different introns. Transcription produces nine different genes, seven variable spliced and two unspliced forms, but only six-spliced mRNAs coding protein. The mRNAs seem different from truncation of the 5’ end, truncation of the 3’ end, existing or missing a cassette exon, splicing and retaining of an intron. There are two possible alternative promoters, two not overlapping alternative last exons and five verified alternative polyadenylation sites[16].

PTN has been confirmed to be upregulated in some types of cancers[2,4–9,13,14] and to play an important part in tumour angiogenesis[2,4] and metastasis[5,9]. Yet, the definit mechanism of PTN in cancer is not completely clear. It has been shown that overexpression of PTN has multiple effects on various cancers through diverse mechanisms. PTN has been considered as a candidate angiogenic factor in breast cancer[17], melanoma[18] and prostate cancer[19]. PTN protein expression has been demonstrated to be related to colorectal cancer differentiation and TNM stage[2]. The high level of PTN is associated with high expression of VEGF-A and is considered as a predictor of poor prognosis[2]. Compared to normal brain tissues, the PTN expression increases in low-grade astrocytomas, but the high PTN expression is not related to increasing malignancy grade[20], proliferation rate, microvascular density and poor overall survival[21]. In a latest study, the levels of PTN mRNA and protein was significantly correlated with high histological grade, low Karnofsky Performance Status score, short time to recurrence and poor overall survival in human glioma[3]. High PTN level is positively related to the stage of disease in non-small and small cell lung cancer and inversely to the reaction to treatment[22].

On the mechanism of PTN expression regulation, there are a large number of upstream and downstream molecules. PTN expression is up-regulated by many growth factors and cytokines implicated in cancer growth, such as tumor necrosis factor-α (TNF-α), epidermal growth factor (EGF), platelet-derived growth factor B, ciliary neurotrophic factor and fibroblast growth factors (FGF) 2 and 10[23]. Interferon beta[24] and interferon gamma[25] up-regulate PTN expression in astrocytes and macrophages respectively through STAT1. PTN levels are affected by 1α,25-dihydroxyvitamin D3[26], progesterone[27] and testosterone[28]. PTN expression is down-regulated by menin[29] and PTEN[30], the angiogenic growth factor VEGF-A18, miR-384[9], miR-143[31], miR-499 as well as miR-1709[32]. PTN has been proved to interact with and influence endothelial and cancer cell functions through many cell surface receptors, such as syndecan-3 in advanced stages of prostate cancer[33], anaplastic lymphoma kinase (ALK) in glioblastoma[34,35], breast cancer[36] and lung cancer[37], receptor protein tyrosine phosphatase beta/zeta (RPTPβ/ζ) in breast cancer[38]. PTN directly interacting with integrins[39], nucleolin[40], neuropilin-1[41] leads to the migration of human endothelial cell.

This study indicated that the expression of PTN was remarkably associated with TNM stage and OS. Meta-analysis revealed that the expression of PTN was not associated with tumor size, LNM, DM and histological grade. These three included studies appeared significantly statistical heterogeneity for LNM and the five studies for histological grade. This heterogeneity might have been a result of several design discrepancies among the studies for LNM, including the criterion of high PTN expression, which was obviously different in Wang et al’s study in 2015. When the outlier study was eliminated from the meta-analysis, there existed no proof of heterogeneity in the remaining studies (P = 0.39), and a meta-analysis demonstrated the LNM was significantly related to the high PTN expression. In the same way, this heterogeneity for histological grade might have been from different tumor types in the Yao et al’s study in 2009. There was no evidence that heterogeneity existed in the left studies when excluding outliers from meta analysis (P = 0.97). Meta-analysis showed that high histological grade was significantly correlated with the high PTN expression. These results showed that the overexpression of PTN was closely relevant to invasive behavior of tumor cells and PTN was a prospective biomarker to forecast the prognosis of tumour patients.

In addition, PTN plays important roles via different pathway in different tumors. Over-expression of PTN in three breast cancer cell models has resulted in increased and rapid cancer growth characterized by extensive remodeling of the microenvironment, including increased angiogenesis and striking increases in mouse protocollagens Ialpha2, IValpha5, and XIalpha1, as well as elastin and matrix metalloproteinase-9[42]. The PTN-RPTPβ/ζ pathway has been also reported to affect phosphorylation and/or activation of numerous targets, such as ALK[43], Fyn and its downstream substrate beta catenin, protein kinase C (PKC) alpha or beta through inhibition of the phosphatase activity in breast cancer. PTN interaction with RPTPβ/ζ leads to dephosphorylation and activation of c-Src and subsequently of ανβ3, FAK, phosphoinositide 3-kinase (PI3K) and ERK1/2 in lung cancer[29] and melanoma cells[44]. Through the N-syndecan/PI3K/Akt/mTORC1 pathway, PTN could promote the expression of the SREBP-1c gene, further facilitating denovo lipogenesis by up-regulating the lipogenic enzyme FAS in hepatocellular carcinoma[9]. In colorectal cancer, PTN binds to RPTRβ/ζ and modulates β-catenin phosphorylation, which is the cause of a higher expression of VEGF-A and higher levels of vascularization[2]. Interestingly, two naturally occurring forms of PTN (18 and 15 kDa) that differ by 12 amino acids at their C-terminal region, differentially promote glioblastoma migration and proliferation. PTN15 promotes glioblastoma proliferation in an ALK-dependent fashion, whereas immobilized PTN18 promotes haptotactic migration of glioblastoma cells in an RPTPβ/ζ-dependent fashion[35], indicating that both ALK and RPTPβ/ζ may be important in this type tumor. Inactivation of the PTN gene with PTN-targeted hammerhead ribozyme constructs has inhibited PTN-induced colony formation and prevents tumor growth in mice, suggesting that PTN may be playing major role in the metastatic growth of melanoma cells[18]. This effect coincides with down-regulation of the cell cycle regulator cyclin E and up-regulation of the cell cycle inhibitor p21WAF1/Cip1[45]. Down-regulation of RPTPβ/ζ expression has been shown to initiate epithelial-tomesenchymal transition and to increase experimental prostate cancer metastasis in nude mice, while the effects of PTN in prostate cancer growth have been attributed to its interaction with syndecan-3[46]. These contribute to the formation of heterogeneity in some cases, such as LNM and histological grade.

The result of this meta analysis should be explained under several important constraints. In evaluating the association between PTN expression and LNM/ histological grade, the heterogeneity detection indicated significant heterogeneity. The heterogeneity might be caused by different tumors and different cut-points of high PTN expression. Moreover, some studies of small sample might also contribute to the formation of heterogeneity. Aslo, there might be different proportion of advanced tumors in different research centers, which could be a cause of heterogeneity.

Secondly, P value in Egger’s test showed significant difference in the TNM stage group. This meant that there was publication bias in the TNM stage group. Bias was mainly due to the inclination of positive publication and the ignore of negative results. Publication bias could only increase the unreliability. In addition, the summary in meetings was excluded, which might lead to publishing bias.

Thirdly, although most data were directly available in research, some studies only offered survival curves, which leaded to possible deviations between estimated and actual statistical data. In order to reduce the deviation as much as possible, detailed steps had been taken.

At last, population primarily came from East Asia and did not well represent the population all over the world.

Generally speaking, this meta-analysis suggests that the high expression of PTN is significantly relevant to advanced TNM stage and poor OS and can serve as a promising biomarker to predict unfavorable survival outcomes. PTN may be a potential target for tumor treatments. Meanwhile, the high expression of PTN is not associated with tumor size, LNM, DM and histological grade. So, larger scale, multicentre and higher quality studies will be needed to verify our results.

## Supporting information

S1 File PRISMA 2009 checklist. (DOC)

## References

[1] Ferlay J, Soerjomataram I, Dikshit R, Eser S, Mathers C, et al (2015) Cancer incidence and mortality worldwide: sources, methods and major patterns in GLOBOCAN 2012. Int J Cancer 136: E359–386. 10.1002/ijc.29210 25220842

[2] Kong Y, Bai PS, Nan KJ, Sun H, Chen NZ, et al (2012) Pleiotrophin is a potential colorectal cancer prognostic factor that promotes VEGF expression and induces angiogenesis in colorectal cancer. Int J Colorectal Dis 27: 287–298. 10.1007/s00384-011-1344-z 22065111

[3] Ma J, Lang B, Wang X, Wang L, Dong Y, et al (2014) Co-expression of midkine and pleiotrophin predicts poor survival in human glioma. J Clin Neurosci 21: 1885–1890. 10.1016/j.jocn.2014.02.020 25001988

[4] Lei Zhang SK, Tjerk Feenstra, Xiujuan Li, Chuan Jin, Liisi Laaniste, Tamador Elsir Abu El Hassan,2 K. Elisabet Ohlin, Di Yu, Tommie Olofsson, Anna-Karin Olsson,3 Fredrik Pontén,1 Peetra U. Magnusson,1 Karin Forsberg Nilsson, Magnus Essand,1 Anja Smits, Lothar C. Dieterich, Anna Dimberg (2015) Pleiotrophin promotes vascular abnormalization in gliomas and correlates with poor survival in patients with astrocytomas. Science Signaling 8: 12. 10.1126/scisignal.aaa1690 26645582

[5] Wu H BA, Babb J, Klein-Szanto A, Godwin A, Elenitsas R, Gelfand JM LS, Seykora JT. (2005) Pleiotrophin expression correlates with melanocytic tumor progression and metastatic potential. Journal of Cutaneous Pathology 32: 5. 10.1111/j.0303-6987.2005.00282.x 15606670

[6] Yao J, Ma Q, Wang L, Zhang M (2009) Pleiotrophin expression in human pancreatic cancer and its correlation with clinicopathological features, perineural invasion, and prognosis. Dig Dis Sci 54: 895–901. 10.1007/s10620-008-0433-5 18716876

[7] Ma J, Kong Y, Nan H, Qu S, Fu X, et al (2017) Pleiotrophin as a potential biomarker in breast cancer patients. Clin Chim Acta 466: 6–12. 10.1016/j.cca.2016.12.030 28041942

[8] WANG HWaJ (2015) Expression of pleiotrophin in small cell lung cancer. J Biol Regul Homeost Agents 29: 4. 25864755

[9] Bai PS, Xia N, Sun H, Kong Y (2017) Pleiotrophin, a target of miR-384, promotes proliferation, metastasis and lipogenesis in HBV-related hepatocellular carcinoma. J Cell Mol Med 21: 3023–3043. 10.1111/jcmm.13213 28557334 PMC5661149

[10] Klomp HJ, Zernial O, Flachmann S, Wellstein A, Juhl H (2002) Significance of the expression of the growth factor pleiotrophin in pancreatic cancer patients. Clin Cancer Res 8: 823–827. 11895915

[11] Tierney JF, Stewart LA, Ghersi D, Burdett S, Sydes MR (2007) Practical methods for incorporating summary time-to-event data into meta-analysis. Trials 8: 16 10.1186/1745-6215-8-16 17555582 PMC1920534

[12] Parmar MK, Torri V, Stewart L (1998) Extracting summary statistics to perform meta-analyses of the published literature for survival endpoints. Stat Med 17: 2815–2834. 9921604 10.1002/(sici)1097-0258(19981230)17:24<2815::aid-sim110>3.0.co;2-8

[13] Dapeng Wu LL, Xuebing Yan3, Chunyan Wang, Yaling Wang, Kun Han, Shuchen Lin, Zhihua Gan and Daliu Min (2017) Pleiotrophin promotes chemoresistance to doxorubicin in osteosarcoma by upregulating P-glycoprotein. Oncotarget 8: 14. 10.18632/oncotarget.19148 PMC5609967 28969035

[14] Moon H-S, Park WI, Sung SH, Choi E-Ah, Chung H-W, et al (2003) Immunohistochemical and quantitative competitive PCR analyses of midkine and pleiotrophin expression in cervical cancer. Gynecologic Oncology 88: 289–297. 12648577 10.1016/s0090-8258(02)00070-7

[15] Rauvala H (1989) An 18-kd heparin-binding protein of developing brain that is distinct from fibroblast growth factors. EMBO J 8: 2933–2941. 2583087 10.1002/j.1460-2075.1989.tb08443.x PMC401361

[16] Papadimitriou E, Pantazaka E, Castana P, Tsalios T, Polyzos A, et al (2016) Pleiotrophin and its receptor protein tyrosine phosphatase beta/zeta as regulators of angiogenesis and cancer. Biochim Biophys Acta 1866: 252–265. 10.1016/j.bbcan.2016.09.007 27693125

[17] Choudhuri R, Zhang HT, Donnini S, Ziche M, Bicknell R (1997) An angiogenic role for the neurokines midkine and pleiotrophin in tumorigenesis. Cancer Res 57: 1814–1819. 9135027

[18] Czubayko F, Schulte AM, Berchem GJ, Wellstein A (1996) Melanoma angiogenesis and metastasis modulated by ribozyme targeting of the secreted growth factor pleiotrophin. Proc Natl Acad Sci U S A 93: 14753–14758. 8962127 10.1073/pnas.93.25.14753 PMC26208

[19] Jager R, Noll K, Havemann K, Pfluger KH, Knabbe C, et al (1997) Differential expression and biological activity of the heparin-binding growth-associated molecule (HB-GAM) in lung cancer cell lines. Int J Cancer 73: 537–543. 9389569 10.1002/(sici)1097-0215(19971114)73:4<537::aid-ijc14>3.0.co;2-6

[20] Ulbricht U, Brockmann MA, Aigner A, Eckerich C, Muller S, et al (2003) Expression and function of the receptor protein tyrosine phosphatase zeta and its ligand pleiotrophin in human astrocytomas. J Neuropathol Exp Neurol 62: 1265–1275. 14692702 10.1093/jnen/62.12.1265

[21] Peria FM, Neder L, Marie SK, Rosemberg S, Oba-Shinjo SM, et al (2007) Pleiotrophin expression in astrocytic and oligodendroglial tumors and it's correlation with histological diagnosis, microvascular density, cellular proliferation and overall survival. J Neurooncol 84: 255–261. 10.1007/s11060-007-9379-2 17443289

[22] R Ja¨ger BL, Knabbe C, Souttou B, Raulais D, Zeiler T, Wellstein A, Aigner A, Neubauer A and Zugmaier G (2002) Serum levels of the angiogenic factor pleiotrophin in relation to disease stage in lung cancer patients. British Journal of Cancer 86: 5 10.1038/sj.bjc.6600006 11953815 PMC2364151

[23] Papadimitriou E, Mikelis C, Lampropoulou E, Koutsioumpa M, Theochari K, et al (2009) Roles of pleiotrophin in tumor growth and angiogenesis. Eur Cytokine Netw 20: 180–190. 10.1684/ecn.2009.0172 20167557

[24] Satoh J, Kuroda Y (2001) Differing effects of IFN beta vs IFN gamma in MS: gene expression in cultured astrocytes. Neurology 57: 681–685. 11524479 10.1212/wnl.57.4.681

[25] Li F, Tian F, Wang L, Williamson IK, Sharifi BG, et al (2010) Pleiotrophin (PTN) is expressed in vascularized human atherosclerotic plaques: IFN-{gamma}/JAK/STAT1 signaling is critical for the expression of PTN in macrophages. FASEB J 24: 810–822. 10.1096/fj.09-140780 19917672 PMC2830133

[26] Tamura M, Ichikawa F, Guillerman RP, Deuel TF, Nodal M (1995) 1alpha,25-Dihydroxyvitamin D(3) down-regulates pleiotrophin messenger RNA expression in osteoblast-like cells. Endocrine 3: 21–24. 10.1007/BF02917444 21153232

[27] Milhiet PE, Vacherot F, Caruelle JP, Barritault D, Caruelle D, et al (1998) Upregulation of the angiogenic factor heparin affin regulatory peptide by progesterone in rat uterus. J Endocrinol 158: 389–399. 9846168 10.1677/joe.0.1580389

[28] Orr B, Vanpoucke G, Grace OC, Smith L, Anderson RA, et al (2011) Expression of pleiotrophin in the prostate is androgen regulated and it functions as an autocrine regulator of mesenchyme and cancer associated fibroblasts and as a paracrine regulator of epithelia. Prostate 71: 305–317. 10.1002/pros.21244 20812209 PMC3045659

[29] Feng SBG Z.J., Wu Y., Xu X.F., Hua X., Jin G.H., (2010) Lung cancer cellmigration is regulated via repressing growth factor PTN/RPTP β/ζ signaling bymenin. Oncogene 29: 5416–5426. 10.1038/onc.2010.282 20639902 PMC3007126

[30] Li G, Hu Y, Huo Y, Liu M, Freeman D, et al (2006) PTEN deletion leads to up-regulation of a secreted growth factor pleiotrophin. J Biol Chem 281: 10663–10668. 10.1074/jbc.M512509200 16507572

[31] Yi C, Xie WD, Li F, Lv Q, He J, et al (2011) MiR-143 enhances adipogenic differentiation of 3T3-L1 cells through targeting the coding region of mouse pleiotrophin. FEBS Lett 585: 3303–3309. 10.1016/j.febslet.2011.09.015 21945314

[32] Lee JY, Jeong W, Lim W, Kim J, Bazer FW, et al (2012) Chicken pleiotrophin: regulation of tissue specific expression by estrogen in the oviduct and distinct expression pattern in the ovarian carcinomas. PLoS One 7: e34215 10.1371/journal.pone.0034215 22496782 PMC3319562

[33] Mikelis C, Koutsioumpa M, Papadimitriou E (2007) Pleiotrophin as a possible new target for angiogenesis-related diseases and cancer. Recent Pat Anticancer Drug Discov 2: 175–186. 18221061 10.2174/157489207780832405

[34] Koyama-Nasu R, Haruta R, Nasu-Nishimura Y, Taniue K, Katou Y, et al (2014) The pleiotrophin-ALK axis is required for tumorigenicity of glioblastoma stem cells. Oncogene 33: 2236–2244. 10.1038/onc.2013.168 23686309

[35] Lu KV, Jong KA, Kim GY, Singh J, Dia EQ, et al (2005) Differential induction of glioblastoma migration and growth by two forms of pleiotrophin. J Biol Chem 280: 26953–26964. 10.1074/jbc.M502614200 15908427

[36] Perez-Pinera P, Chang Y, Astudillo A, Mortimer J, Deuel TF (2007) Anaplastic lymphoma kinase is expressed in different subtypes of human breast cancer. Biochem Biophys Res Commun 358: 399–403. 10.1016/j.bbrc.2007.04.137 17490616 PMC1945107

[37] Gao SB, Feng ZJ, Xu B, Wu Y, Yin P, et al (2009) Suppression of lung adenocarcinoma through menin and polycomb gene-mediated repression of growth factor pleiotrophin. Oncogene 28: 4095–4104. 10.1038/onc.2009.273 19749796 PMC5399538

[38] Meng K, Rodriguez-Pena A, Dimitrov T, Chen W, Yamin M, et al (2000) Pleiotrophin signals increased tyrosine phosphorylation of beta beta-catenin through inactivation of the intrinsic catalytic activity of the receptor-type protein tyrosine phosphatase beta/zeta. Proc Natl Acad Sci U S A 97: 2603–2608. 10.1073/pnas.020487997 10706604 PMC15975

[39] Mikelis C, Sfaelou E, Koutsioumpa M, Kieffer N, Papadimitriou E (2009) Integrin alpha(v)beta(3) is a pleiotrophin receptor required for pleiotrophin-induced endothelial cell migration through receptor protein tyrosine phosphatase beta/zeta. FASEB J 23: 1459–1469. 10.1096/fj.08-117564 19141530

[40] Koutsioumpa M, Drosou G, Mikelis C, Theochari K, Vourtsis D, et al (2012) Pleiotrophin expression and role in physiological angiogenesis in vivo: potential involvement of nucleolin. Vasc Cell 4: 4 10.1186/2045-824X-4-4 22423616 PMC3379939

[41] Elahouel R, Blanc C, Carpentier G, Frechault S, Cascone I, et al (2015) Pleiotrophin exerts its migration and invasion effect through the neuropilin-1 pathway. Neoplasia 17: 613–624. 10.1016/j.neo.2015.07.007 26408254 PMC4674489

[42] Chang Y, Zuka M, Perez-Pinera P, Astudillo A, Mortimer J, et al (2007) Secretion of pleiotrophin stimulates breast cancer progression through remodeling of the tumor microenvironment. Proc Natl Acad Sci U S A 104: 10888–10893. 10.1073/pnas.0704366104 17578909 PMC1904160

[43] Deuel TF (2013) Anaplastic lymphoma kinase: "Ligand Independent Activation" mediated by the PTN/RPTPbeta/zeta signaling pathway. Biochim Biophys Acta 1834: 2219–2223. 10.1016/j.bbapap.2013.06.004 23777859

[44] Gao SB, Feng ZJ, Xu B, Chen Y, Zheng HH, et al (2011) Menin represses malignant phenotypes of melanoma through regulating multiple pathways. J Cell Mol Med 15: 2353–2363. 10.1111/j.1582-4934.2010.01222.x 21129151 PMC3822947

[45] Satyamoorthy K, Oka M, Herlyn M (2000) An antisense strategy for inhibition of human melanoma growth targets the growth factor pleiotrophin. Pigment Cell Res 13 Suppl 8: 87–93. 11041363 10.1034/j.1600-0749.13.s8.16.x

[46] Diamantopoulou Z, Kitsou P, Menashi S, Courty J, Katsoris P (2012) Loss of receptor protein tyrosine phosphatase beta/zeta (RPTPbeta/zeta) promotes prostate cancer metastasis. J Biol Chem 287: 40339–40349. 10.1074/jbc.M112.405852 23060448 PMC3504749

